# Chorea me a river: depression in Huntington’s disease as an exemplar of precision medicine

**DOI:** 10.1093/braincomms/fcac294

**Published:** 2022-11-09

**Authors:** Anthony J Hannan

**Affiliations:** Florey Institute of Neuroscience and Mental Health, University of Melbourne, Parkville, VIC 3010, Australia; Department of Anatomy and Physiology, University of Melbourne, Parkville, VIC 3010, Australia

## Abstract

This scientific commentary refers to ‘Different depression: motivational anhedonia governs antidepressant efficacy in Huntington’s disease’ by McLauchlan *et al.* (https://doi.org/10.1093/braincomms/fcac278).

**This scientific commentary refers to ‘Different depression: motivational anhedonia governs antidepressant efficacy in Huntington’s disease’ by McLauchlan *et al.*** (https://doi.org/10.1093/braincomms/fcac278).

Huntington’s disease is a neurodegenerative disorder caused by a tandem-repeat (CAG-trinucleotide) DNA expansion encoding an extended polyglutamine tract in the huntingtin protein. Despite the fact that Huntington’s disease was first described by George Huntington in 1872 (exactly 150 years prior to publication of the present article) and that it has been almost 30 years since the discovery of the causative tandem-repeat gene mutation was published, there are still no disease-modifying therapies available for this fatal disorder.^1^ In addition to cognitive deficits (culminating in dementia) and motor dysfunction (e.g. chorea), psychiatric symptoms are prominent, the most common of which is depression.^2^ However, considering how devastating depression, and other psychiatric and cognitive symptoms, can be for Huntington’s disease family members, they have surprisingly not received the same attention as chorea and other motor symptoms.

Depression occurs in approximately one-third to three quarters of clinical Huntington’s disease populations, which is much higher than the prevalence in the general population (i.e. those without the Huntington’s disease gene mutation). And yet our understanding of depression in Huntington’s disease, including evidence for the most efficacious approaches to treat this specific population, is rudimentary at best.^2^ It was argued by some that the increased prevalence of depression in Huntington’s disease was due to psychosomatic factors associated with the knowledge of being at risk of a fatal disease. However, the first demonstration that preclinical animal models of Huntington’s disease exhibited both face and predictive validity for clinical depression,^3,4^ despite the fact that these transgenic mice (unlike Huntington’s disease family members) could not be aware that they expressed the Huntington’s disease gene mutation, provided clear evidence that depression is intrinsic to this neurodegenerative disease, rather than a psychosomatic manifestation. The fact that the transgenic mice exhibited depressive-like behaviours, which not only responded to antidepressant drugs but also exercise,^3–5^ demonstrates that the Huntington’s disease gene mutation, and associated cascade of molecular and cellular changes in the brains of the mice, is driving these depression-like changes. It should be noted that in this mouse model of Huntington’s disease, other depression-like molecular and cellular changes have been found, including neurotrophic and serotonergic dysregulation, hypothalamic–pituitary–adrenal dysfunction and deficits of hippocampal neurogenesis.^2–5^ Furthermore, the fact that clinical diagnosis of depression is not completely penetrant in those with the Huntington’s disease gene mutation is presumably due to genetic and environmental modifiers, with clear evidence of gene–environment interactions provided by transgenic Huntington’s disease mice.^4,5^

A new article in *Brain Communications*^6^ provides novel insights regarding depression, motivational anhedonia and antidepressant efficacy in Huntington’s disease. In this study, McLaughlan and colleagues^6^ made use of an exceptionally valuable international clinical research platform, ENROLL-HD, to establish which drugs are most effective for depression in Huntington’s disease. ENROLL-HD, which has been generously funded by the CHDI Foundation, has over 21 000 participants internationally, including gene-positive pre-symptomatic and symptomatic individuals, and is the world’s largest observational study of HD families.

These investigators^6^ studied 5486 gene-positive adult patients in ENROLL-HD receiving antidepressant medication. The outcome measures included standard clinical depression scales at first follow-up (the primary outcome) and all follow-ups (the secondary outcome) and the intervention was defined as the class of antidepressants prescribed. It was found, for the primary outcome, that selective serotonin-reuptake inhibitors (SSRIs) were superior to serotonin–noradrenaline reuptake inhibitors for depression in Huntington’s disease. The secondary outcome was that both SSRIs and bupropion (a norepinephrine–dopamine reuptake inhibitor) were more effective than serotonin–noradrenaline reuptake inhibitors.^6^ SSRIs and bupropion have also been shown to exhibit efficacy in ameliorating depressive-like behaviours in transgenic Huntington’s disease mice,^3–5,7^ further supporting the strong construct, face and predictive validity of this preclinical model.

A second study conducted by McLaughlan and colleagues^6^ was on a much smaller scale, involving recruitment of 51 gene-positive adult patients and 26 controls. These investigators used a cognitive battery based on the Research Domain Criteria for Depression, a framework that aims to complement traditional psychiatric assessments. In this study, the authors found evidence that depression in Huntington’s disease may be specifically associated with motivational anhedonia (measured as reduced effort for reward) and is not explained by apathy.^6^ This provides further evidence that depression in Huntington’s disease is not identical to depression in the general population. One implication is that depression in Huntington’s disease could be diagnosed with different (or at least additional) criteria and, together with the evidence from the first study, that it should be treated differently from depression in the general population. The authors link the two studies, noting that bupropion has been found to ameliorate motivational anhedonia and exhibits a synergistic effect when co-administered with SSRIs.

The numbers of participants means that the first study, in the large ENROLL-HD cohort, provides a higher level of evidence, and thus the efficacy of different antidepressants for depression in Huntington’s disease represent the key findings of this article.^6^ However, the second study^6^ provides important insights into the nature of depression, motivational anhedonia and other affective and cognitive aspects of Huntington’s disease, which should be followed-up in larger independent cohorts.

These findings firstly have implications for the treatment of depression in those who are gene-positive for the Huntington’s disease mutation, whether or not they are motor symptomatic (i.e. clinically diagnosed with Huntington’s disease neurological symptoms). Rather than treating depression in Huntington’s disease based on the assumption that it is identical to depression in the general (non-Huntington’s disease) population, this clinical challenge may require a precision medicine approach. The greatly increased incidence of depression in Huntington’s disease may not only mean that the pathogenic mechanisms, as well as the clinical manifestation, are different from depression in the general (non-Huntington’s disease) population, but also that its treatment may need to incorporate strategies of precision medicine which involve mechanistic approaches to disease biomarkers and clinical stratification. Furthermore, if depression in Huntington’s disease is viewed in a new light, as a potentially unique subclass of depression, then tailored treatments may not only increase efficacy, but also reduce side-effects, and associated suffering.

We can take this argument further and use depression in Huntington’s disease as an exemplar of precision medicine (Fig. 1). Those with a fully penetrant tandem-repeat expansion in the *huntingtin* (*HTT*) gene are destined to develop the disease, unless an intervention can be developed to prevent or delay onset. As well as this Huntington’s disease gene mutation, each individual has the remaining approximately 3 billion base pairs of unique DNA in their unique genome (or ‘semi-unique’ in the case of identical twins), some of which may either increase their predisposition, or resilience, to depression. However, depression is also the result of complex gene–environment interactions, and therefore the ‘envirome’^8^ of the individual (their entire environmental exposures and experience throughout life) will influence whether they develop depression at a given stage in life (Fig. 1). Thus, whilst the Huntington’s disease gene mutation adds to the genetic load for depression (one might imagine it fills the ‘genetic predisposition bucket’ further) there is a requirement for additional environmental exposures (e.g. stress) to cause the ‘spill-over’ into clinical depression. Additionally, it cannot be assumed that this depression will be identical to that observed in the general population, but rather it may have some Huntington’s disease-specific features.

**Figure 1:**
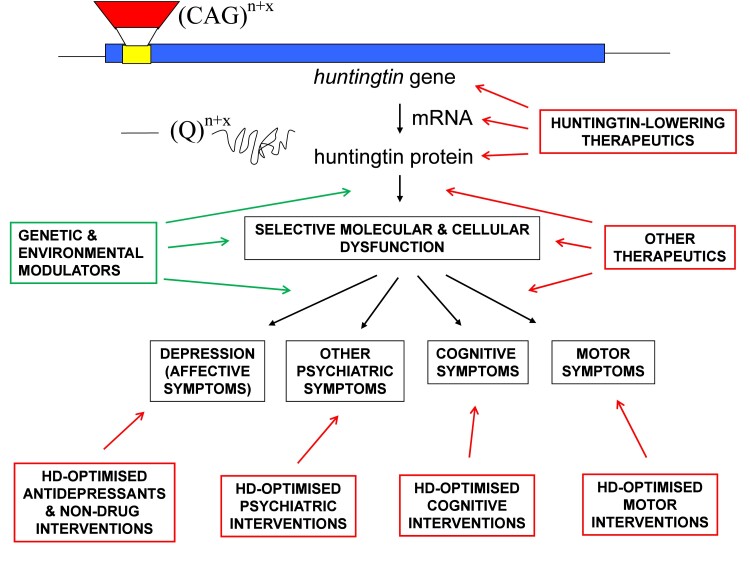
**Diagram schematically illustrating pathogenic pathways of Huntington’s disease and how precision medicine approaches could be applied to Huntington’s disease.** Huntington’s disease is caused by a trinucleotide (CAG) repeat expansion (‘*n* + x’ represents the number (*n*) of tandem repeats in the normal range plus the extra (x) CAG repeats associated with the Huntington’s disease gene mutation). The *huntingtin* gene is transcribed into mRNA which is then translated to produce huntingtin protein, with an expanded tract of glutamine (Q) amino acids (a Q^n + x^ polyglutamine tract) encoded by the CAG repeat expansion mutation. The complex cascades of molecular and cellular pathogenesis are simplified, in the interests of clarity. Depression is the most common psychiatric manifestation of Huntington’s disease, although other psychiatric symptoms can also occur. Cognitive symptoms are common, and motor symptom onset is used for clinical (neurological) diagnosis of Huntington’s disease in gene-positive individuals. There are many potential preventative and therapeutic approaches that could be applied to Huntington’s disease. An obvious approach, which is being actively investigated, involves huntingtin-lowering therapeutics, which may be targeted at DNA, RNA and/or protein levels (including targeting somatic cells with CRISPR-mediated gene editing, antisense oligonucleotides, etc.). A range of other therapeutic options may have potential efficacy via targeting downstream molecular and cellular components of pathogenic pathways. However, considering the heterogeneity and complexity of symptoms, many therapeutic interventions will continue to target specific psychiatric (e.g. depression), cognitive and motor symptoms. Precision medicine, based on detailed mechanistic understanding of Huntington’s disease pathogenesis at molecular, cellular and systems levels, will help improve the lives of families impacted by this devastating disorder. It should be noted that, again in the interests of simplicity and clarity, the peripheral (‘non-brain’) symptoms of Huntington’s disease (e.g. those symptoms associated with hypothalamic–pituitary–adrenal axis and gastrointestinal dysfunction) have not been addressed in this diagram but are nevertheless clinically significant and may require their own precision medicine approaches. Furthermore, these potential interventions (noting that there are currently no disease-modifying treatments for Huntington’s disease clinically available) would not all be applied to an individual, but would be stratified to disease stage (e.g. huntingtin-lowering strategies are likely to have to be administered very early to be effective) and individual characteristics (e.g. genomic and other biomarker data, outside the Huntington’s disease gene mutation, could facilitate pharmacogenomics and other precision medicine approaches to maximize efficacy and minimize side-effects), including patient-specific combinations of symptoms. Finally, polypharmacy may be required for some individuals, and different pharmacological and non-drug interventions may be attempted at progressive stages of Huntington’s disease, as part of a long-term strategy to prevent, treat and eventually cure this devastating disease.

One additional consideration regarding the nature of depression in Huntington’s disease is the increasing evidence that Huntington’s disease is not simply a brain disease but rather a systemic disease of brain and body. A striking demonstration of this peripheral pathology in Huntington’s disease, with major implications for peripheral modulation of brain function, is the evidence that gut microbiota are dysregulated (i.e. dysbiosis occurs) in both this preclinical mouse model^9^ and clinical Huntington’s disease.^10^ Considering the evidence that the microbiota–gut–brain axis may modulate affective function, including that associated with depression, these brain–body interactions in Huntington’s disease may be highly relevant to such psychiatric manifestations.^2^

Depression is also a common psychiatric feature in other neurodegenerative disorders, including Alzheimer’s disease and Parkinson’s disease. Thus, this kind of precision medicine approach could be applied to other neurodegenerative diseases. Rather than assume that depression in Alzheimer’s disease and Parkinson’s disease is identical to depression in the general population, the similarities and differences should be systematically investigated, at the level of pathogenic mechanisms, disease biomarkers and clinical stratification. Similarly, the relative efficacy of different antidepressant interventions in Alzheimer’s disease and Parkinson’s disease should also be explored on a large scale, so that precision medicine approaches can also be applied to these other neurodegenerative diseases, in the same manner outlined for Huntington’s disease (Fig. 1).

Huntington’s disease is one of the most extraordinary, and devastating, of all human disorders. Its autosomal dominant nature means that it strikes, on average, every second child of an affected parent. The complex combination of psychiatric, cognitive, motor and peripheral symptoms, together with the current absence of effective disease-modifying therapies, make it extremely difficult to manage clinically. And yet there is much cause for hope. The collective power of multiple fields of science, including genetics, biochemistry, cell biology and neuroscience, place us on the cusp of novel therapeutic breakthroughs. However, rather than conveniently avoid the complexity that links molecules to mind in such neurological and psychiatric disorders, we need to confront these complex pathogenic mechanisms head-on (whilst not forgetting the role of bidirectional brain–body interactions), applying the power of computational biology and integrative neuroscience to deliver novel approaches for prevention and treatment.

## Data Availability

Data sharing is not applicable to this article as no new data were created or analysed.
